# Effect of Functional Nanoporous TiO_2_ Film Obtained on Ti6Al4V Implant Alloy to Improve Resistance in Biological Solution for Inflammatory Conditions

**DOI:** 10.3390/ijms24108529

**Published:** 2023-05-10

**Authors:** Lidia Benea, Anca Ravoiu Lupu, Iulian Bounegru, Petrica Vizureanu

**Affiliations:** 1 Competences Center: Interfaces-Tribocorrosion-Electrochemical Systems, Dunarea de Jos University of Galati, 47 Domnească Street, RO-800008 Galati, Romania; iulian.bounegru@ugal.ro; 2 Faculty of Medicine and Pharmacy, Dunarea de Jos University of Galati, 35 Al. I. Cuza Street, RO-800010 Galati, Romania; 3 Faculty of Materials Science and Engineering, “Gheorghe Asachi” Technical University of Iasi, RO-700050 Iasi, Romania

**Keywords:** biomaterials, biologic fluid, inflammatory compound, reactive oxygen species, corrosion

## Abstract

The metallic titanium-based biomaterials are sensitive to corrosion-induced degradation in biological fluids in the presence of inflammatory conditions containing reactive oxygen species (ROS). Excess ROS induces oxidative modification of cellular macromolecules, inhibits protein function, and promotes cell death. In addition, ROS could promote implant degradation by accelerating the corrosive attack of biological fluids. The functional nanoporous titanium oxide film is obtained on titanium alloy to study the effect on implant reactivity in biological fluid with reactive oxygen species such as hydrogen peroxide, which are present in inflammations. The TiO_2_ nanoporous film is obtained by electrochemical oxidation at high potential. The untreated Ti6Al4V implant alloy and nanoporous titanium oxide film are comparatively evaluated for corrosion resistance in biological solution by Hank’s and Hank’s doped with hydrogen peroxide by electrochemical methods. The results showed that the presence of the anodic layer significantly improved the resistance of the titanium alloy to corrosion-induced degradation in biological solutions under inflammatory conditions.

## 1. Introduction

In recent years, there has been a substantial focus on the development and application of advanced materials in the biomedical domain, particularly in the context of implantable devices and tissue engineering. However, the term “biomaterial” can meet different interpretations both in materials science and clinical medicine. Hence, a biomaterial is characterized as a synthetic material designed to replace a component of a biological system or to serve a specific function in direct contact with living tissue. This definition highlights the critical role that biomaterials play in the development of advanced medical devices and implants, necessitating a thorough understanding of their interactions with biological systems and their ability to perform effectively within a physiological environment. A biomaterial is distinct from a biological material, such as bone, which originates within a living system. Biomaterials are classified according to their structural, chemical, and biological characteristics, for example, metallic, ceramic, polymeric, and composite with varying degrees of bioactivity [1]. Biomaterials represent a field of interdisciplinary research that requires sufficient knowledge from three different fields, such as (i) materials science and engineering (the interrelationship between processing-structure properties of synthetic and biological materials, including metals, ceramics, polymers, composites, and tissues); (ii) biology, cell physiology, molecular biology, anatomy, animal, and human physiology; (iii) clinical sciences dentistry, ophthalmology, orthopedics, plastic and reconstructive surgery, cardiovascular surgery, neurosurgery, immunology, histopathology, experimental surgery, medicine, and veterinary surgery [1].

Research conducted to date has determined that among metallic biomaterials, titanium alloys exhibit the greatest biocompatibility, corrosion resistance, and specific strength (the ratio of tensile strength to density) when compared to stainless steels and CoCr alloys. CoCr alloys, on the other hand, display superior wear resistance and relatively higher strength in comparison to stainless steel and titanium-based alloys [2]. The Ti6Al4V alloys are used as structural biomaterials for the manufacture of orthopedic prostheses and dental implants due to their excellent properties of specific strength, corrosion resistance, and biocompatibility [3].

The biological environment is surprisingly aggressive and can lead to the rapid or gradual degradation of many implant materials [4]. Superficially, one might think that the neutral pH, low salt content, and modest body temperature would constitute a milder environment. Cells that secrete enzymes and strong oxidizing agents are directed to damage the material [5]. Subsequently, strong degradation agents are concentrated between the cell and the implanted material [6]. Moreover, during inflammatory conditions, reactive species are generated. From a clinical point of view, inflammation is characterized by redness, warmth, swelling, sensitivity, and loss of function in the affected organ [7]. At the cellular level, it is characterized by tissue damage associated with a local accumulation of inflammatory leukocytes, including neutrophils, eosinophils, macrophages, lymphocytes, and platelets [8]. An inflammatory response represents an essential defense mechanism against the invasion of the body by microorganisms and elements that it considers foreign [9].

When O_2_ is metabolized (used for cellular respiration), a reduction reaction takes place, resulting in the formation of a radical, the main reactive oxygen species (ROS) formed by respiratory cells. The reduction in molecular oxygen (O_2_) leads to the formation of superoxide ($O_{2}^{-}$), precursor compound of many reactive oxygen species. It is unreactive, as in its presence $O_{2}^{-}$ is transformed to form the much more reactive hydrogen peroxide ($H_{2}O_{2}$). Hydrogen peroxide ($H_{2}O_{2}$) is also an endogenous product that appears as a response to inflammatory reactions after a transplant [10] and during wound healing as inflammatory cells release hydrogen peroxide into the extracellular environment to defend themselves against foreign bodies [10].

As inflammation is part of the complex biological response of body tissues to harmful stimuli affecting the chemical environment in an organism in different percentages [11], its influence on the implant must be evaluated over time. Corrosion or tribocorrosion of the Ti6Al4V implant alloy in biological solutions is well studied in the specialized literature [12,13,14,15,16], as is the beneficial effect of different methods of forming the titanium oxide film on it [17,18]. On the contrary, only scant information is related to the long-time influence of oxygen-reactive species such as hydrogen peroxide on Ti alloy implants. Other authors discuss the fabrication and analysis of a Ti6Al4V implant for cranial restoration [19].

This research paper emphasizes the impact of the electrochemically obtained TiO_2_ nanoporous film on the Ti6Al4V implant alloy, aimed at enhancing its resistance to the corrosive effects of Hank’s biological solution under inflammatory conditions. In other words, it focuses on the presence of reactive oxygen species (ROS), such as hydrogen peroxide, during inflammation.

Thus, the polarization resistance and corrosion rate of the untreated grade 5 Ti6Al4V implant alloy and the same alloy with the electrochemically formed nanoporous titanium oxide film on its surface in Hank’s biological solution and the biological solution doped with the existing compound in inflammatory conditions, or oxygen reactive species (ROS), respectively, hydrogen peroxide, are studied and comparatively evaluated.

## 2. Results and Discussion

### 2.1. Monitoring the Evolution of Open Circuit Potential (OCP) during Immersion Time

The grade 5 titanium alloy, Ti6Al4V, is considered the best choice for permanent implants. This can be attributed to its biocompatibility, osseo-integrating property, high strength/weight ratio, higher corrosion resistance, non-magnetic properties, high hardness, good mechanical strength, etc. [20,21].

From the point of view of the test solution, Hank’s solution is chosen, simulating the human body’s blood [19]. The initial interactions of blood with an endosseous implant can influence clot formation, eventual migration, and the differentiation of osteogenic cells in the healing compartment. As the migration of osteogenic cells is the hallmark of post-condition, both the formation of a fibrin scaffold and the activation of blood cells trapped at the implant interface could play a key role in this first stage of the peri-implant bone healing period. Thus, an understanding of the establishment of the blood/implant interface may be of considerable importance in understanding the early mechanisms of peri-implant healing [22].

The open-circuit potential (OCP) method, or evolution of free potential vs. time, is the starting point for all types of electrochemical experiments to study the corrosion phenomenon [20]. The open-circuit potential method is a more qualitative than quantitative method for determining the behavior of a material in the corrosion process in a certain environment and shows its tendency to oxidation or passivation in an electrochemical environment [20]. OCP is a mixed potential determined by the oxidation and reduction reactions that take place at the surface of a metal electrode [20,23].

**Figure 1:**
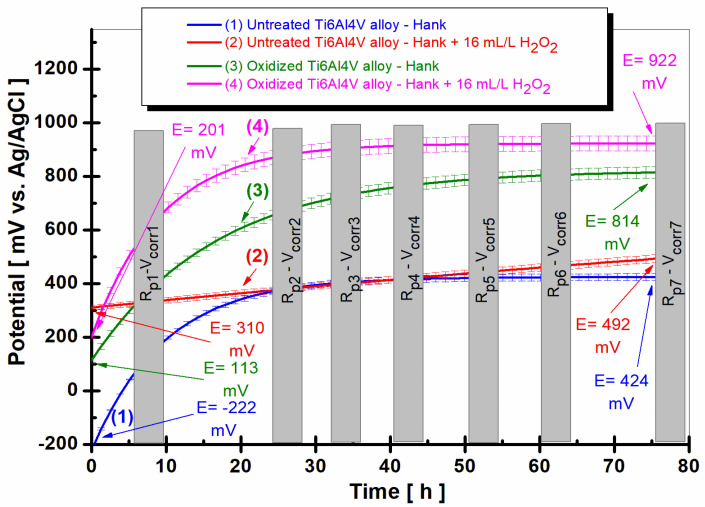
The comparative evolution of the free potential of the untreated and electrochemically oxidized grade 5 Ti6Al4V alloy immersed in the 2 solutions: (1) untreated Ti6Al4V alloy immersed in Hank’s solution; (2) untreated Ti6Al4V alloy immersed in Hank’s solution doped with H_2_O_2_; (3) oxidized Ti6Al4V alloy immersed in Hank’s solution; (4) oxidized Ti6Al4V alloy immersed in Hank’s solution doped with H_2_O_2_.

Figure 1 shows the evolution of the free potential of the untreated and electrochemically oxidized grade 5 Ti6Al4V alloy immersed for a period of 75.5 h in Hank’s solution and Hank’s solution doped with hydrogen peroxide. To provide a better understanding of the relationship between free potential and corrosion behavior at the molecular level, we will discuss the underlying molecular processes corresponding to the observed changes in free potential.

The free potential (OCP) observed in Figure 1 is the result of the balance of oxidation and reduction reactions occurring on the titanium alloy surface. These reactions involve electron transfer between the metal surface and the surrounding environment, and their rates determine the overall free potential of the system.

For the untreated titanium alloy immersed in Hank’s solution, the initially negative potential suggests a higher rate of oxidation reactions (corrosion) at the metal surface. These reactions involve the transfer of electrons from the metal surface to the surrounding solution, leading to the dissolution of the metal ions [20]. As the potential shifts to more positive values, the rate of reduction reactions (passivation) increases. This increase indicates the formation of a protective oxide layer, such as TiO_2_, on the alloy surface [24]. This layer reduces the corrosion rate by acting as a barrier between the metal and the corrosive environment, leading to a more stable potential over time.

In the case of the untreated titanium alloy in Hank’s solution doped with hydrogen peroxide, the initially higher potential indicates a lower corrosion rate due to the passivating effect of the H_2_O_2_ [24]. Hydrogen peroxide can react with the titanium surface to promote the growth of a TiO_2_ layer, which contributes to the passivation of the surface [24,25,26,27,28]. The gradual shift to even more positive values suggests further passivation of the alloy surface, which could be attributed to the competing processes of TiO_2_ growth and dissolution in the presence of hydrogen peroxide [24,25,26,27,28]. As hydrogen peroxide can also dissolve the oxide layer, the dynamic equilibrium between TiO_2_ growth and dissolution may result in the observed free potential changes [24,25,26,27,28].

Elemental analysis of oral mucosa around Ti and Ti alloy dental implants has demonstrated increased release of Ti from implant surfaces in inflamed sites compared to healthy tissues, although the exact mechanisms underlying the observation have not been determined [23]. During inflammation, H_2_O_2_ levels can become elevated because they are produced directly by bacteria (at levels that can exceed 5 mM) or by immune cells that have migrated to the inflamed site [29,30]. The corrosion resistance of both untreated and oxidized surfaces in Hank’s solution doped with hydrogen peroxide is also studied to study the influence of hydrogen peroxide as an inflammatory compound.

The oxidized titanium alloy immersed in Hank’s solution as well as in Hank’s doped with hydrogen peroxide solution starts from the beginning of immersion time with more positive values of open-circuit potential, 113 mV vs. Ag/AgCl (curve 3) and 201 mV vs. Ag/AgCl, respectively, in curve (4). The open-circuit potential of oxidized titanium alloy increases slowly with immersion time until around 60 h, after being constant and revealing at the end of monitoring a positive value of 814 mV vs. Ag/AgCl. The potential difference between the oxidized and untreated alloys after 75.5 h of immersion in Hank’s solution is ∆E = 390 mV.

The most positive (noble) potential is recorded for the oxidized titanium alloy immersed in Hank’s solution doped with hydrogen peroxide, having a value of 201 mV vs. Ag/AgCl, which moves towards even more positive (noble) values after immersion up to about 30 h, after which the value of the free potential remains approximately constant at 922 mV vs. Ag/AgCl, curve (4). Thus, the potential difference between the untreated and oxidized alloy immersed in Hank’s solution doped with hydrogen peroxide becomes even greater, having a value of ∆E = 430 mV. This behavior could be explained by the dual role of hydrogen peroxide in promoting the growth and dissolution of TiO_2_ on the surface of the titanium alloy [24,25,26,27,28]. 

For the untreated alloy in the presence of hydrogen peroxide (curve 2), the initially more positive potential indicates a lower corrosion rate, which could be attributed to the passivating effect of H_2_O_2_-promoted TiO_2_ growth [24]. However, the simultaneous dissolution of the oxide layer by hydrogen peroxide creates a dynamic equilibrium between the oxide layer growth and dissolution processes [24,25,26,27,28], leading to the observed gradual shift of the potential to more positive values. Similarly, for the oxidized alloy in the presence of hydrogen peroxide (curve 4), the initially higher potential indicates better corrosion resistance due to the pre-existing oxide layer, with the continuous shift to more positive values suggesting further passivation of the alloy surface because of the competing processes of TiO_2_ growth and dissolution in the presence of hydrogen peroxide [24,25,26,27,28]:

(1) $$Ti+H_{2}O_{2}\to TiO_{2}+H_{2}$$

(2) $$2H_{2}O_{2}\to 2H_{2}O+O_{2}$$

(3) $$TiO_{2}+2H_{2}O\to Ti{\left(OH\right)}_{4}$$

(4) $$TiO_{2}+nH_{2}O\to TiO_{2}nH_{2}O$$

(5) $$(Ti-OH)+H_{2}O\to {\left[Ti-O\right]}^{-}+H_{3}O^{+}$$

(6) $$TiO_{2}nH_{2}O+OH^{-}\to HTiO_{3}^{-}nH_{2}O$$

Titanium present in a titanium alloy can react with hydrogen peroxide, resulting in the formation of a titanium oxide film, as shown in Equation (1). Moreover, some of the TiO_2_ could be hydrolyzed (see Equations (2) and (3)). A hydration reaction, Equation (4), occurs simultaneously with the formation of Ti-OH functional groups, Equation (5). Hydrated and hydrolyzed titanium oxide (TiO_2_) can cause the formation of negatively charged surfaces (Equations (5) and (6)), which could be involved in the increase in the free potential to a more negative value during the immersion time, followed by the transition to a more positive value, as explained by Equation (1) [24].

### 2.2. Evaluation of Polarization Resistance (R_p_) of the Untreated Grade 5 Ti6Al4V Alloy and the Electrochemically Oxidized Alloy under the Influence of Hydrogen Peroxide in Biological Solution

Another direct current electrochemical method is the evaluation of polarization resistance (R_p_) and corrosion rate (V_corr_) from linear polarization (PL) curves. Linear polarization can be used to characterize the electrolyte-material pair by scanning the potential-current (i-E) domain over a narrow (linear) potential domain, where Tafel slopes can be plotted. On this narrow range in the vicinity of the corrosion potential, the obtained current response is linear [20,21].

The polarization resistance method involves obtaining multiple linear polarization curves and calculating the polarization resistance and corrosion rate using the Stern–Geary equation.

In the case of this research work, it is considered that the measurement of one hundred polarization curves is necessary to have 100 value points for the polarization resistance and 100 value points for the corrosion rate, respectively. Hence, each polarization resistance measurement sequence in the experimental protocol has 100 value points.

The polarization resistance (R_p_) is defined as the variation of the potential relative to the variation of the current in the considered linear range, as follows:

(7) $$R_{p}=\frac{\Delta E}{\Delta i}$$

The Stern–Geary equation was used to calculate corrosion current density as follows:

(8) $$i_{cor}=\frac{B}{R_{p}}$$

(9) $$B=\frac{b_{a}\left|b_{c}\right|}{2.303\left(b_{a}+b_{c}\right)}$$

(10) $$i_{corr}=\frac{b_{a}\left|b_{c}\right|}{2.303\left(b_{a}+b_{c}\right)}/R_{p}$$

where:

i_corr_ is the corrosion current density.
B—specific constant for each system: material/environment.
b_a_ anodic Tafel slope.
b_c_ the cathodic Tafel slope.

**Figure 2:**
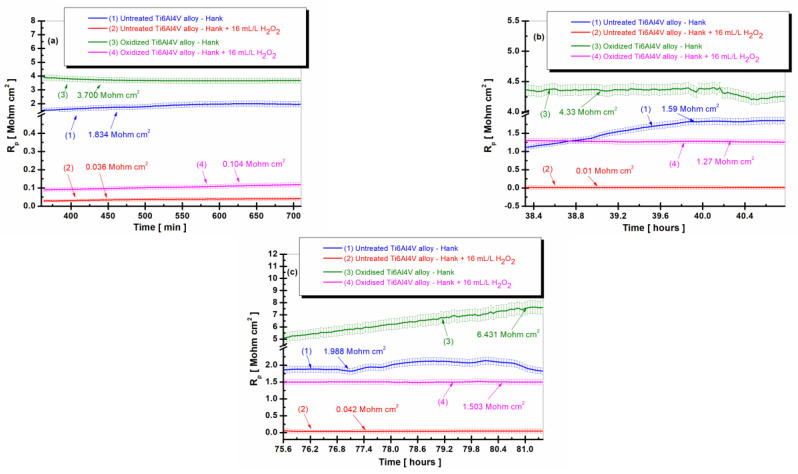
The comparative evolution of the polarization resistance during the immersion period in Hank’s biological solution and Hank’s doped with 16 mL/L H_2_O_2_: (**a**) after 6 h (360 min); (**b**) after 38 h (2298 min); (**c**) after 75.5 h (4534 min): (1) untreated Ti6Al4V alloy in Hank’s biological solution; (2) untreated Ti6Al4V alloy in Hank’s doped with hydrogen peroxide; (3) oxidized titanium alloy in Hank’s biological solution; and (4) oxidized titanium alloy in Hank’s doped with hydrogen peroxide.

The comparative evolution of the polarization resistance for the untreated alloy and for the oxidized alloy with a titanium oxide film (TiO_2_) is presented in Figure 2a–c at three times the total immersion period of the samples in Hank’s biological solution and Hank’s doped with 16 mL/L H_2_O_2_.

Thus, after 6 h of immersion, in Figure 2a, the polarization resistance (R_p_) of the untreated titanium alloy in the Hank’s solution has a value of 1.834 Mohm·cm^2^, curve (1), while the polarization resistance of the electrochemically oxidized titanium alloy presents constant values with stabilization at a value of 3700 Mohm·cm^2^ at the end of the first measurement period, curve (3), being thus higher than the polarization resistance for the untreated alloy. 

In the case of Hank’s biological solution doped with hydrogen peroxide, the polarization resistance of the untreated titanium alloy drops drastically to the value of 0.036 Mohm·cm^2^, being approximately fifty times lower as compared with the value from Hank’s solution, curve (2). For the electrochemically oxidized alloy, there is also a decrease in the polarization resistance after 6 h, R_p_ = 0.104 Mohm·cm^2^, with curve (4) being approximately 10 times higher than that of the untreated titanium alloy in the presence of the inflammatory compound, curve (2), where R_p_ = 0.036 Mohm·cm^2^.

After 38 h of immersion, as shown in Figure 2b, the polarization resistance value of the untreated grade 5 Ti6Al4V alloy in Hank’s solution at the end of the measurement period is 1.59 Mohm·cm^2^, lower than in the first measurement period, as shown in curve (1).

The values of the polarization resistance of the electrochemically oxidized Ti6Al4V alloy after 38 h in Hank’s solution are slightly higher than in the first measurement period, with R_p_ having a value of 4.33 Mohm·cm^2^ towards the end of the measurement period. Thus, even after 38 h, the R_p_ value for the electrochemically oxidized titanium alloy is approximately three times higher than the R_p_ value of the untreated titanium alloy, which confirms the effectiveness of the electrochemical oxidation process of the titanium alloy for improving the implant’s resistance to the aggressive action of biological fluids in the human body.

Furthermore, after 38 h of immersion (Figure 2b), the value of the polarization resistance of the untreated grade 5 Ti6Al4V alloy immersed in Hank’s biological solution doped with H_2_O_2_ at the end of the measurement period is 0.010 Mohm·cm^2^, curve (2), lower than in the first measurement period. The polarization resistance values of the electrochemically oxidized Ti6Al4V alloy after 38 h in Hank’s biological solution doped with H_2_O_2_ are slightly higher than in the first measurement period, with R_p_ having a value of 1.275 Mohm·cm^2^ towards the end of the measurement period, curve (4). Thus, even after 38 h, the R_p_ value for the electrochemically oxidized titanium alloy is approximately 127 times higher than the R_p_ value of the untreated titanium alloy, which confirms the effectiveness of the electrochemical oxidation process of the titanium alloy for improving the implant’s resistance to the aggressive action of the inflammatory compound in the biological fluid.

After 75.5 h of immersion, as shown in Figure 2c, in Hank’s and Hank’s biological solutions doped with H_2_O_2_, the same trend of the polarization resistance values is preserved for the two surfaces, the untreated immersed Ti6Al4V alloy and the electrochemically oxidized Ti6Al4V alloy. The value of the polarization resistance (R_p_) of the untreated alloy in Hank’s solution increases slightly to R_p_ = 1.988 Mohm·cm^2^, as shown in curve (1), and in Hank’s solution doped with H_2_O_2_ to R_p_ = 0.042 Mohm·cm^2^. The values of the polarization resistance of the electrochemically oxidized alloy remain higher compared to those of the untreated alloy during this measurement period as well, with R_p_ being R_p_ = 6.431 Mohm·cm^2^ in Hank’s solution and R_p_ = 1.503 Mohm·cm^2^ in Hank’s solution doped with H_2_O_2_.

**Figure 3:**
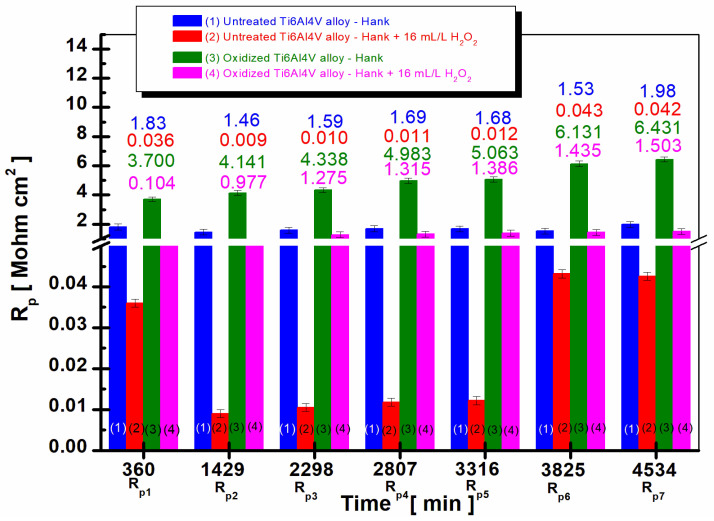
The comparative evolution of the mean values of polarization resistance during the immersion period in Hank’s biological solution and Hank’s doped with 16 mL/L H_2_O_2_: (1) untreated Ti6Al4V alloy in Hank’s biological solution; (2) untreated Ti6Al4V alloy in Hank’s doped with hydrogen peroxide; (3) oxidized titanium alloy in Hank’s biological solution; and (4) oxidized titanium alloy in Hank’s doped with hydrogen peroxide.

For a better highlighting of the results obtained regarding the evaluation of the polarization resistance throughout the immersion period in the two solutions, Figure 3 presents the average values (the mean values) of all the polarization resistance values recorded at different immersion periods for the untreated Ti6Al4V alloy compared to the Ti6Al4V alloy electrochemically oxidized.

Figure 3 shows that in the case of electrochemically oxidized grade 5 Ti6Al4V alloy immersed in Hank’s solution, columns (3), and in the Hank’s solution doped with H_2_O_2_, columns (4), the polarization resistance values are higher compared to the values recorded for the untreated Ti6Al4V alloy, columns (1) and (2), at all times studied during the entire immersion period.

At the molecular level, the observed trends in polarization resistance can be understood by considering the surface reactions and oxide layer formation on the titanium alloy. For the untreated alloy immersed in Hank’s solution (1), the lower polarization resistance values can be attributed to the native oxide layer present on the alloy surface, which offers limited protection against corrosion. However, when the untreated alloy is immersed in Hank’s solution doped with H_2_O_2_ (2), the addition of hydrogen peroxide promotes the growth of a TiO_2_ layer while also causing its dissolution. This dynamic equilibrium between the oxide layer growth and dissolution processes leads to lower polarization resistance values, indicating a higher corrosion rate for the untreated alloy in the presence of H_2_O_2_.

On the other hand, for the electrochemically oxidized Ti6Al4V alloy, the presence of a thicker TiO_2_ layer offers improved protection against corrosion, as evidenced by the higher polarization resistance values observed in both Hank’s solution (3) and Hank’s solution doped with H_2_O_2_ (4). The continuous increase in polarization resistance values with immersion time for the oxidized alloy can be attributed to the further growth and passivation of the TiO_2_ layer on the alloy surface, which enhances the alloy’s resistance to the aggressive action of biological fluids and the inflammatory compounds present in the solutions.

In addition, in the case of the electrochemically oxidized Ti6Al4V alloy, an increase in the values of the polarization resistance is observed with the increase in immersion time. If, at the first measurement, the electrochemically oxidized alloy has a value of R_p1_ = 0.104 Mohm·cm^2^, at the end of the 80 h monitoring, it reaches a value of R_p7_ = 1.503 Mohm·cm^2^, a behavior that demonstrates the effectiveness of the electrochemical oxidation process of the titanium alloy for improving the resistance of the implant to the aggressive action of biological fluids. 

The same trend is observed in Hank’s solution doped with H_2_O_2_ for the electrochemically oxidized titanium alloy, increasing the polarization resistance with the immersion time. At the first measurement, column (3), R_p1_ = 3.700 Mohm·cm^2^ at the end of 80 h immersion, the polarization resistance value increases to R_p7_ = 6.431 Mohm·cm^2^. The increase in the polarization resistance values in the case of the electrochemically oxidized titanium alloy is due to the presence of the TiO_2_ layer, which has superior insulating properties than that of native titanium oxide formed on the untreated alloy.

If for the electrochemically oxidized Ti6Al4V alloy, an increase in the polarization resistance values is observed with increasing immersion time, for the untreated Ti6Al4V alloy, it is observed that the polarization resistance values are lower and show a slight decrease after 38 h of immersion compared to the value obtained after 360 min and a very slight increase at the end of the immersion period for both Hank’s biological solution and Hank’s doped with H_2_O_2_, columns (1) and (2). The observed behavior results from the non-uniform native titanium oxide film on the untreated alloy surface, which accelerates degradation compared to electrochemically oxidized titanium alloy counterparts.

### 2.3. Evaluation of the Corrosion Rate (V_corr_) of the Untreated Ti6Al4V Alloy and the Electrochemically Oxidized Alloy

The corrosion rate calculated according to Equation (10) is expressed as corrosion current density (with units of measurement, for example, mA/cm^2^) and can be converted into penetration index (with units of measurement, for example, mm/an) with the help of Faraday’s laws, considering the oxidation reaction, the equivalent weight (electrochemical equivalent, μ_eq_) and the valence of the respective metal, n. The electrochemical equivalent for pure elements is the ratio between the atomic weight (u.a.m) and the number of electrons transferred in the oxidation reaction:

(11a) $$\mu_{eq}=\frac{M}{n}$$

where:

M—atomic mass.
n—the number of transferred electrons.

The electrochemical equivalent of a substance denotes the mass of that substance accumulated on one of the electrodes as a 1 ampere current flows through it for a 1 s duration. In this case, the formula for finding electrochemical equivalents is as follows:

(11b) $$\mu_{eq}=\frac{M}{q}=\frac{M}{I\cdot t}$$

where M is the mass of the substance and q is the charge passed. q = I t, where I is the applied current and t is the time.

The electrochemical equivalent of titanium is about sixteen for the reduction reaction in an acidic medium and about 12 for the oxidation reaction.

For alloys, the electrochemical equivalent is obtained by adding the electrochemical equivalent of each element in the alloy.

The charge transferred during an electrochemical process is related to the amount of material passing through the corrosion half-reaction of R species (a metallic material under the corrosion process) by the following equation:

(12) $$R\Leftrightarrow Qx^{n+}+ze$$

The change in reactant mass, R, is related to the current flow in Equation (12). The relationship is given by Faraday’s Law, where Q is the charge (in units of C-coulombs) resulting from the electrolytic reaction in Equation (12), z is the number of electrons transferred per reaction, F is Faraday’s constant (96.485 C/mol) and N is the number of moles of R that have gone through the reaction in Equation (13). Faraday’s law is the basis for converting corrosion current density to penetration index and corrosion rate to mass loss.

(13) Q=zNF

The charge, Q, can be defined in terms of electric current, where i is the current (in units of A) and t is the duration (in units of s) of the electrolysis of the specie(s) R, as in Equation (14).

(14) $$Q=\int_{0}^{t}idt$$

By combining Equations (13) and (14) and rearranging, one can determine the number of moles of material, N, that react (corrode) each time (Equation (15)).

(15) $$N=\frac{\int_{0}^{t}idt}{nF}$$

By using Faraday’s Law in this form, the basic equation for determining corrosion rates is obtained. To calculate the corrosion rate from the point of view of the penetration rate (penetration index), V_corr(ip)_, the definitions of i_corr_ and Faraday’s Law are shown in Equations (8)–(15), respectively, and the density of the metal (in units of g/cm^3^) and a proportionality constant, K_p_, are shown in Equation (16). The penetration rate can be calculated in several useful units, given the correct proportionality constant.

(16) $$V_{corr(ip)}=\mu_{eq}\frac{i_{cor}}{\rho }$$

where μ_eq_—electrochemical equivalent; ρ—metal density in g/cm^3^.

**Figure 4:**
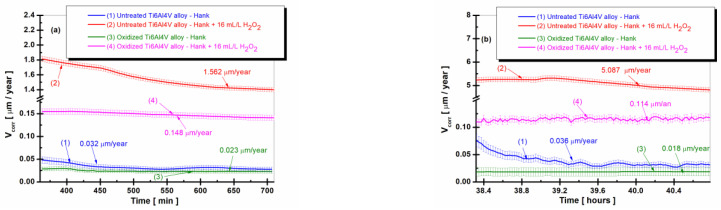

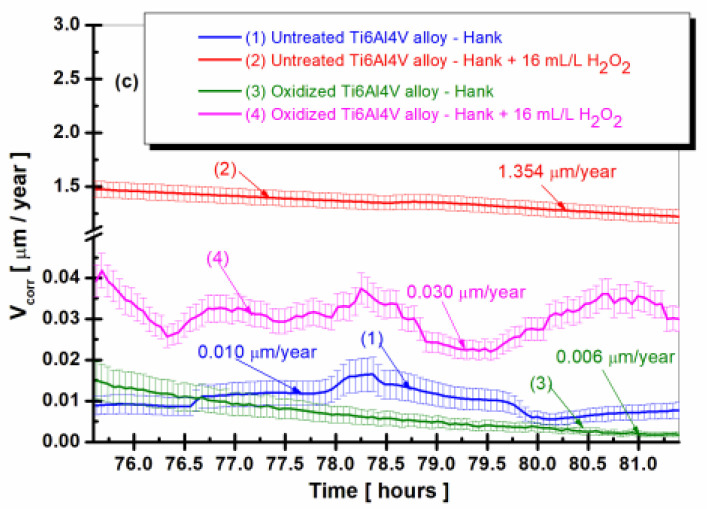
The comparative evolution of the corrosion rate during the immersion period in Hank’s biological solution and Hank’s doped with 16 mL/L H_2_O_2_: (**a**) after 6 h (360 min); (**b**) after 38 h (2298 min); (**c**) after 75.5 h (4534 min): (1) untreated Ti6Al4V alloy in Hank’s biological solution; (2) untreated Ti6Al4V alloy in Hank’s doped with hydrogen peroxide; (3) oxidized titanium alloy in Hank’s biological solution; and (4) oxidized titanium alloy in Hank’s doped with hydrogen peroxide.

**Figure 5:**
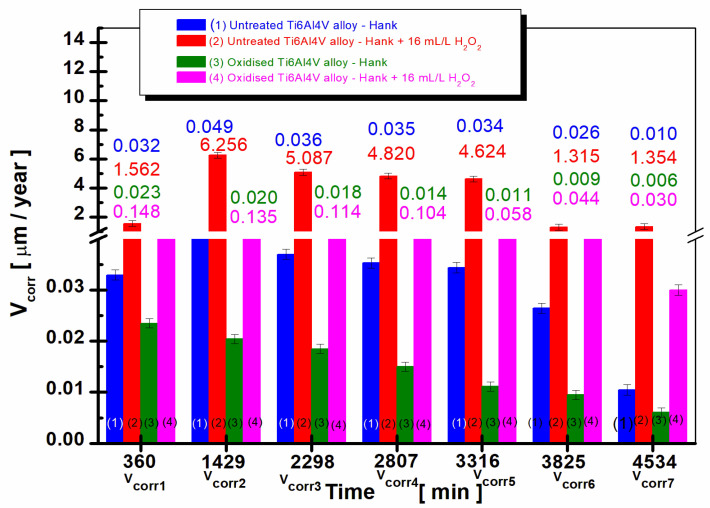
The comparative evolution of the mean values of corrosion rate during the immersion period in Hank’s biological solution and Hank’s doped with 16 mL/L H_2_O_2_: (1) untreated Ti6Al4V alloy in Hank’s biological solution; (2) untreated Ti6Al4V alloy in Hank’s doped with hydrogen peroxide; (3) oxidized titanium alloy in Hank’s biological solution; and (4) oxidized titanium alloy in Hank’s doped with hydrogen peroxide.

The variation of the corrosion rate expressed as a penetration index for the untreated alloy and for the oxidized alloy having the titanium oxide (TiO_2_) film on its surface is shown in Figure 4a–c at three times the total immersion period of the samples immersed in Hank’s biological solution and Hank’s doped with H_2_O_2,_ and in Figure 5 as an average value at all times studied during the entire immersion period.

From Figure 4a–c, it can be seen that the values of the corrosion rate (V_corr_) for the untreated Ti6Al4V alloy immersed in Hank’s biological solution and Hank’s solution doped with H_2_O_2_, curves (1) and (2), are higher than the corrosion rate values recorded for the electrochemically controlled oxidized Ti6Al4V alloy having the titanium oxide (TiO_2_) film formed on its surface for all measurement periods during immersion. Thus, after 6 h of immersion (Figure 4a), the corrosion rate (V_corr_) of the untreated titanium alloy immersed in Hank’s solution doped with H_2_O_2_ has a slight decrease from immersion, with stabilization at a value of 1.562 µm/year (curve (2)) having the highest corrosion rate. The value of the corrosion rate of the electrochemically oxidized titanium alloy shows constant values with stabilization at a value of 0.148 µm/year at the end of the first measurement period (curves (4)), having a ten times lower value than that of the untreated alloy. After 38 h of immersion, in Figure 4b, the corrosion rate values of the untreated Ti6Al4V alloy show a slight increase and oscillations, so that at the end of this measurement period, the value of V_corr_ is 5.087 µm/year, higher than in the first measurement period.

The corrosion rate values of the electrochemically oxidized Ti6Al4V alloy after 38 h are slightly lower than in the first measurement period, with V_corr_ having a value of 0.114 µm/year towards the end of the measurement period. Consequently, even after 38 h, the corrosion rate (V_corr_) for the electrochemically oxidized titanium alloy is 44 times lower than that of the untreated titanium alloy. This result underscores the effectiveness of the electrochemical oxidation process in enhancing the implant’s resistance to the aggressive action of biological fluids within the human body. 

Thus, even after 38 h, the V_corr_ for the electrochemically oxidized titanium alloy is 44 times lower than the V_corr_ for the untreated titanium alloy, which confirms the effectiveness of the electrochemical oxidation process of the titanium alloy for improving the resistance of the implant to the aggressive action of biological fluids in the human body.

After 75.5 h of immersion in both Hank’s biological solution and Hank’s solution containing H_2_O_2_, Figure 4c shows that the corrosion rate values for both the untreated Ti6Al4V alloy and the electrochemically oxidized Ti6Al4V alloy follow a similar trend in the two solutions. The corrosion rate (V_corr_) of the untreated alloy immersed in Hank’s solution with H_2_O_2_ experiences a slight decrease towards the end of the immersion period, reaching V_corr_ = 1.354 µm/year, as shown in curve (2). Meanwhile, the corrosion rate of the electrochemically oxidized Ti6Al4V alloy also decreases, reaching V_corr_ = 0.030 µm/year by the end of the immersion period, as depicted in the curve (4), which is approximately 45 times lower.

From the comparative analysis of the average values of the corrosion rate (Figure 5) it is observed that in the case of the electrochemically oxidized grade 5 Ti6Al4V alloy immersed in Hank’s solution and in Hank’s solution doped with H_2_O_2_, the corrosion rate values are lower compared to the untreated grade 5 Ti6Al4V alloy at all the times studied. Moreover, in the case of the electrochemically oxidized Ti6Al4V alloy, a decrease in the corrosion rate values is observed with increase in immersion time (columns (3) and (4) from Figure 5).

If, at the first measurement, the electrochemically oxidized alloy immersed in Hank’s solution doped with H_2_O_2_ has a value of V_corr1_ = 0.148 µm/year, at the end of the 80 h monitoring, it reaches a value of V_corr7_ = 0.030 µm/year, a behavior that demonstrates the effectiveness of the electrochemical oxidation process on the titanium alloy to improve the corrosion resistance of the implant to the aggressive action of the biological fluids in the human body and the presence of the inflammatory compound.

In the case of the untreated Ti6Al4V alloy, it is observed that the corrosion rate values show the highest values both in the Hank’s solution and the Hank’s solution doped with H_2_O_2_, columns (1) and (2). Further, in the case of untreated alloys, the corrosion rate values tend to increase slightly. 

After 38 h of immersion, V_corr2_ for the untreated alloy in Hank’s solution increases to V_corr2_ = 0.049 µm/year compared to the value obtained after 360 min, V_corr1_ = 0.032 µm/year. The corrosion rate values for the untreated alloy immersed in Hank’s solution doped with H_2_O_2_ increase much more over time. Thus, after 38 h, V_corr2_ is 6256 µm/year compared to the average value recorded after 360 min of V_corr1_ = 1562 µm/year.

At the molecular level, the corrosion velocity profiles for each group can be explained by considering the interactions between the titanium alloy surface and the immersion solutions. In the case of untreated Ti6Al4V alloy immersed in Hank’s solution doped with H_2_O_2_ (2), the increase in corrosion velocity can be attributed to the dynamic equilibrium between the growth and dissolution of the TiO_2_ layer on the alloy surface, promoted by the presence of hydrogen peroxide. This dynamic equilibrium implicates a higher corrosion rate, as the oxide layer does not provide complete protection against corrosion. However, as the immersion time progresses, the dissolution rate of the TiO_2_ layer may slow down due to saturation effects in the solution, leading to a gradual increase in the protective oxide layer thickness. This increase in thickness results in a decrease in corrosion velocity after reaching the maximum value. It is important to note that even with the observed decrease, the corrosion rate for the untreated alloy in the H_2_O_2_-doped solution remains significantly higher than the other groups, indicating an increased susceptibility to corrosion in the presence of inflammatory compounds.

### 2.4. Morphological, Compositional, and Structural Investigation

**Figure 6:**
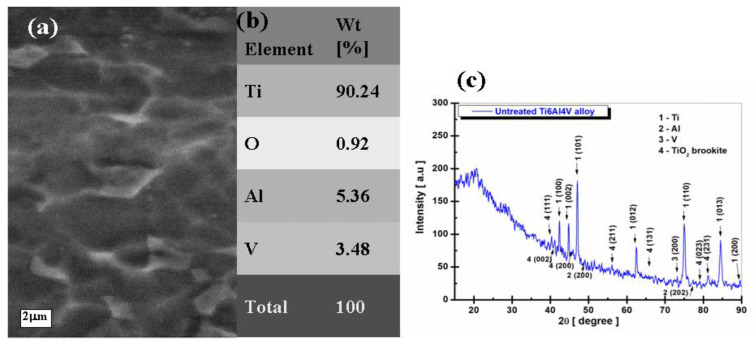
Morphological, compositional, and structural characterization of untreated Ti6Al4V alloy: (**a**) SEM surface morphology; (**b**) EDX elemental composition; and (**c**) XRD pattern.

The morphological, compositional (SEM-EDX), and structural (XRD) analyses were carried out with the help of the pieces of equipment described in Section 3. Figure 6a–c shows the following: (a) SEM surface morphology of the untreated grade 5 Ti6Al4V implant alloy, (b) EDX elemental analysis, and (c) X-ray diffraction pattern.

The SEM-EDX analysis of the untreated Ti6Al4V implant alloy, Figure 6a,b, shows a typical uniform morphology of the titanium alloy surface and indicates the presence of the main elements in the studied alloy, respectively: Ti, Al, and V, with their respective percentage values of Ti = 90.24%, Al = 5.36%, and V = 3.48%. Oxygen is present because a thin layer of titanium oxide with a percentage of 2.29% TiO_2_ is natively formed on the surface of the untreated alloy.

The percentage of TiO_2_ formed on the surface of the Ti6Al4V alloy is determined from the transformation of the mass percentage of oxygen (0.92%) from the general analysis with the help of the molecular mass of TiO_2_ (79.866 g/mol). 

From the surface morphology of the untreated Ti6Al4V alloy (Figure 6), the surface is smooth and has no surface defects.

From the analysis of the XRD spectrum before the electrochemical oxidation process (Figure 6c), the following crystalline phases are identified using the software Match! 3, version 2022, (1) titanium (Ti) with crystallographic planes: (100), (002), (101), (012), (110), (013), and (200), corresponding to 2θ angles = 42.33°, 44.68°, 47.17°, 62.61°, 74.90°, 84.49°, and 89.20°. This detected phase is registered in the database of the program used, Crystallography Open Database (COD) 96-901-6191, and belongs to the hexagonal crystallization system, space group P63/mmc. It was also identified as (2) aluminum (Al) with the crystallographic planes: (200) and (202), corresponding to the angles 2θ = 49.00° and 76.86°, identified with the database of the program COD 96-431-3211, as belonging to the cubic crystallization system, space group F m − 3 m.

Another phase identified is (3) vanadium (V) with the crystallographic plane (200), corresponding to the angles 2θ = 74.12° recorded in the database of the Crystallography Open Database (COD) program 96-410-5684 as belonging to the cubic crystallization system, space group I m − 3 m.

The last phase identified is (4) titanium dioxide (TiO_2_) brookite with crystallographic planes: (111), (002), (211), (131), (231), (023), and (200), corresponding to the angles 2θ = 40.24°, 41.03°, 55.93°, 67.44°, 81.30°, 78.82°, and 45.87°, which belong to the orthorhombic crystallization system, space group (P b c n) registered in the COD 96 database-153-0027.

**Figure 7:**
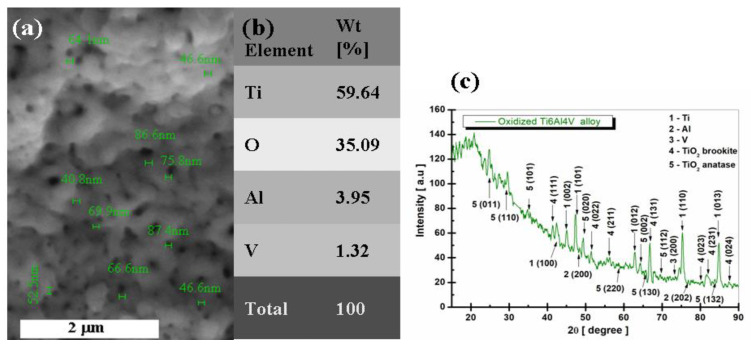
Morphological, compositional, and structural characterization of electrochemically oxidized Ti6Al4V alloy: (**a**) SEM surface morphology and nanopore diameter measurement, (**b**) EDX elemental composition, (**c**) XRD pattern.

Figure 7 shows the morphological, compositional (SEM-EDX), and structural (XRD) analysis of the electrochemically oxidized Ti6Al4V alloy.

From the analysis of Figure 7, the surface of the electrochemically oxidized grade 5 titanium alloy shows a layer of nanoporous, dense TiO_2_, in some places with agglomerations of formed oxide, but without interruptions or surface defects, thus confirming that the voltage and time imposed on the oxidation process are optimal. To highlight the growth of nanopores of TiO_2_ on the surface of the titanium alloy, Figure 7a shows a series of measurements of the diameter of the nanopores, which is located between 40.2 nm and 87.4 nm. The average size of the nanopores obtained at these electrochemical oxidation parameters would be 64 nm.

From the analysis of Figure 7b, the presence of the main elements in the studied biomaterial can be observed, respectively: Ti, Al, and V, with their weight percentage values (wt %), respectively, Ti = 66.18%, Al = 6.01%, and V = 3.23%. At the same time, it is observed that there is an increase in the percentage of oxygen, O = 35.09%, compared to the untreated Ti6Al4V alloy, which confirms the increase of the TiO_2_ layer in a proportion of 87.57% on the surface of the electrochemically oxidized sample.

From Figure 7c, it is observed that the diffraction angles and phases identified for Ti, Al, V, and TiO_2_ are preserved, as in the case of the untreated titanium alloy. In addition to these phases identified in the structural analysis of the untreated titanium alloy, it was noted that in the samples anodically oxidized at 200 V, the crystalline phase of TiO_2_ anatase appeared, identified with the database COD 96-900-8215, which belongs to the tetragonal crystallization system, space group I 41/a m d (141), with the crystallographic planes (011), (110), (101), (020), (220), (002), (130), (112), and (132) corresponding to the diffraction angles 2θ = 24.73°, 29.60°, 34.86°, 49.41°, 58.73°, 64.24°, 65.55°, 69.48 s and 83.76°.

As can be seen from Figure 7c, on the analyzed electrochemically oxidized Ti6Al4V samples, titanium dioxide (TiO_2_ anatase) appeared with significant diffraction peaks on the XRD pattern. This proves that the surface of the titanium alloy oxidizes and forms a passive TiO_2_ anatase film.

From the XRD diagrams in Figure 6c and Figure 7c, it is observed that TiO_2_ anatase electro-crystallizes preferentially according to the planes (011), (110), (020), and (002), which have the highest intensity. It is also observed that at the used oxidation time of 3 min, the peak of TiO_2_ anatase with the crystallization plane (011) appears, a peak that does not appear in the untreated alloy, which proves that more titanium oxide (TiO_2_) is formed on the surface. These data agree with results obtained by other authors on titanium or other titanium alloys [31,32,33,34,35,36,37,38,39,40,41].

It should be noted that studies have been reported in the specialized literature stating that at voltages between 1–130 V applied to the anodic oxidation process, the titanium dioxide formed on the surface of titanium or titanium alloys is amorphous. From 130 V to 250 V, the crystalline phase called anatase is formed [31,32,33,34,35,36,37,38,39,40]. Additionally, Diamnati and his research team [31] state that sulfuric acid is an optimal electrolyte when the final goal of anodization is to obtain anatase.

## 3. Materials and Methods

For this work, grade 5 titanium alloy (Ti6Al4V) purchased from Goodfellow SARL, Lille, France, was used. The purchased plates of Ti6Al4V with a size of 80 × 40 mm and a thickness of 2 mm have the chemical composition and mechanical properties shown in Table 1.

For the anodic oxidation and corrosion tests, the samples were cut to a size of 40 × 40 mm, and by insulating with epoxy resin, a well-defined active surface of 9.6 cm^2^ was established.

**Figure 8:**
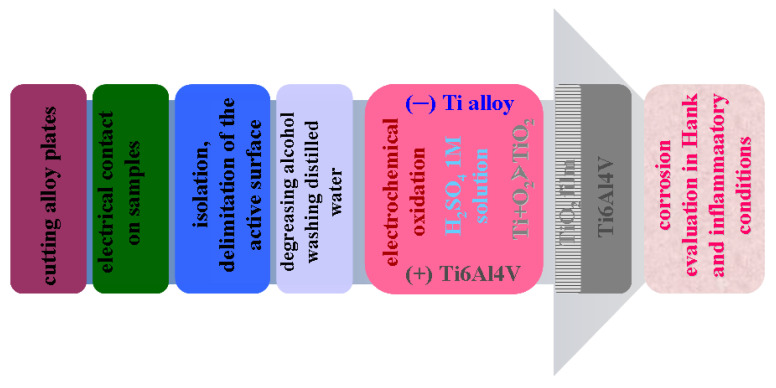
Schematic representation of the processing steps of samples subjected to anodic oxidation.

The electrochemical cell for anodic oxidation was of the two-electrode type. To have electrical contact with the samples, a copper wire was attached to the anode (Ti6Al4V working electrode). Before each experiment, the anode or alloy sample to be oxidized was cleaned with ethanol for 5 min in the ultrasonic bath, after which it was rinsed with distilled water. The steps of sample processing and electrochemical oxidation are described in Figure 8. A Ti6Al4V plate having a higher geometrical surface of 55 cm^2^ was also used as the counter electrode, subjected to the same surface preparation as applied in the case of an anode sample. The volume of H_2_SO_4_ 1M electrolyte in the electrochemical cell was 150 mL with a solution pH of 0.41.

Several voltage values were applied during the anodic oxidation process, in the range from 100 to 250 V, as well as various periods between 0.5 and 4 min. Based on these initial experiments, a voltage of 200 V for 3 min. was selected as an optimized parameter to form anodic titania layers. All oxidation tests were performed at room temperature (22 °C ± 1 °C) and repeated 6 times to check data reproducibility. The mean diameter of nanopores obtained on TiO_2_ film was 64 nm, resulting from the mean value of 10 measurements on an SEM micrograph.

For the evaluation of corrosion resistance, untreated titanium alloy samples and electrochemically oxidized samples were also prepared with an electrical contact, isolating the surfaces to delimit an exact, measurable active surface. The most commonly used electrochemical cell for evaluating the corrosion resistance of materials is the three-electrode cell, which consists of a working electrode (WE), which is the material being studied, a reference electrode (RE), which is the saturated Ag/AgCl electrode with a constant potential of 199 mV vs. the normal hydrogen electrode (EHN) and an auxiliary electrode or counter electrode (CE), which is a chemically inert material consisting of the platinum grid.

The chemical composition of Hank’s solution is shown in Table 2. The chemicals used for the preparation of the solution are of analytical purity and purchased from the Lachner company. The solution was prepared and stored in hermetically sealed vials that have a capacity of 5 L, precisely to keep all the parameters of the solution constant throughout the experimental period.

Hank’s biological solution parameters are determined using the Consort C533 multiparameter, and the results are summarized in Table 3.

For each corrosion resistance evaluation test, a 150 mL of electrolyte was used.

An analytical-grade hydrogen peroxide solution of 30% concentration was used to prepare the simulated inflammatory extreme body environment at 16 mL/L.

From the moment of immersing the sample in the biological solution or the biological solution doped with an inflammatory compound, sequences of electrochemical measurements of free potential (OCP) and polarization resistance (R_p_)-corrosion rate (V_corr_) were performed for 81 h, following measurement sequences as follows:

- Open circuit potential (OCP) at times 0 (immersion), 709 min, 1578 min, 2447 min, 2956 min, 3465 min, and 4174 min.
- Polarization resistance (R_p_) − Corrosion rate (V_corr_) at times 360 min, 1429 min, 2298 min, 2807 min, 3316 min, 3825 min, and 4534 min.

The total duration of immersion of a sample in the biological solution was given by the average duration of an inflammatory process in the human body, which was approximately 3 days.

Each R_p_ − V_corr_ measurement sequence contains 100 value points obtained from as many linear polarization curves in the ±50 mV linear range around the corrosion potential. Linear polarization can be used to characterize the electrolyte-material pair by scanning the potential-current (i-E) domain over a narrow (linear) potential domain, where Tafel slopes can be plotted. On this narrow range in the vicinity of the corrosion potential, the obtained current response is linear [20,21]. The polarization resistance method consists of measuring several linear polarization curves and calculating the polarization resistance and corrosion rate by applying the Stern–Geary equation.

For the structural characterization of both the untreated titanium alloy and the nanoporous film of titanium oxide obtained by electrochemical oxidation, X-ray diffraction (XRD) was used on Dron 3 equipment with an X-ray source of Co Kα radiation (λKa = 1.790300 Ǻ). It was used to analyze and identify crystalline compounds using the Brag–Brentano method. For the interpretation of the spectra, the software Match! 3 correlated with the standard database Crystallography Open Database (COD) was used, the crystalline phases being identified with a code composed of 9 digits. Each analyzed sample was exposed to XRD analysis for 3 s, on a range of 15–90°, at a voltage of 35 kV with an intensity of 20 mA, the measurement step being 0.02°/s.

The morphological and compositional characterization of the surfaces of the untreated titanium alloy and the titanium oxide film (TiO_2_) was performed by scanning electron microscopy (SEM-EDX) using an FEI QUANTA200 electron microscope connected to an EDAX GENESIS X-ray scattered analysis system for the compositional analysis of surfaces. The studied samples were covered with a fine layer of gold to avoid excessive loading of the surfaces due to the presence of oxides that are insulators. The diameter of the nanopores formed was measured directly on the SEM micrographs.

## 4. Conclusions

This research paper highlights the effect of the electrochemical oxidation of the grade 5 Ti6Al4V implant alloy on its behavior during the corrosion degradation process in Hank’s biological solution and Hank’s solution doped with the inflammatory compound hydrogen peroxide over an immersion period of 80 h, which corresponds to the period of an inflammatory process in the human body.

The oxidized titanium implant alloy immersed in Hank’s solution as well as in Hank’s doped with hydrogen peroxide solution starts from the beginning of immersion time with more positive (noble) values of open-circuit potential as compared with untreated titanium alloy.

The open-circuit potential difference between the oxidized alloy and the untreated alloy after 75.5 h of immersion in Hank’s solution is about 390 mV, while the difference in open-circuit potential between the untreated alloy and the oxidized alloy immersed in Hank’s solution doped with hydrogen peroxide becomes even greater, increasing to 430 mV. This behavior denotes a much better behavior of the oxidized alloy compared to the untreated alloy in the presence of the inflammatory product from the biological solution.

Through the comparative analysis conducted to evaluate polarization resistance, it has been demonstrated that the highest values were observed for the electrochemically oxidized titanium alloy immersed in Hank’s biological solution, both in the absence and presence of hydrogen peroxide. This finding confirms the positive influence of the anodic oxide layer on the titanium alloy surface, which contributes to improving the implant’s resistance against the corrosive effects of bodily fluids and inflammatory compounds.

From the comparative analysis of the values of corrosion rate (V_corr_), it is observed that the lowest values of corrosion rate are recorded by the electrochemically oxidized titanium alloy in both studied solutions. This behavior demonstrates that the addition of hydrogen peroxide increases the cathodic and anodic reactions by dissolving the TiO_2_ layer on the surface of the material, thus decreasing the corrosion resistance of the untreated Ti6Al4V alloy.

This behavior confirms the effectiveness of the electrochemical oxidation process of the titanium alloy in improving the resistance of the implant to the aggressive action of the fluids in the human body.

The SEM analysis shows a change in the surface morphology after applying the electrochemical oxidation process. At the same time, the size of the TiO_2_ nanopores can be evaluated from the morphology of the electrochemically oxidized alloy.

From the EDX analysis of the untreated and electrochemically oxidized Ti6Al4V implant alloy, an increase in the mass percentage of oxygen after electrochemical oxidation is observed.

From the structural analysis (XRD), after the electrochemical oxidation, the peak corresponding to the Ti element decreases and there is an increase in the intensity of the TiO_2_ anatase peaks, a behavior that confirms the growth of the TiO_2_ layer through the process of anodic oxidation on the surface of the studied titanium alloy.

Following these results, we can state that the electrochemical modification of the titanium alloy, (Ti6Al4V, through the anodic oxidation method) confirms the possibility of improving its anticorrosive properties for the applicability of the implant in biological solution and in the presence of the inflammatory compound.

## Figures and Tables

**Table 1:** Chemical composition and mechanical properties of the Ti6Al4V alloy.

Specificații	8-12-05832-1	N	Al	C	V	H	Fe	O	Ti
Grade 5 Ti6Al4V	Max. (%)	0.003	6.01	0.008	3.83	0.002	0.083	0.088	89.976
	Min. (%)	0.003	5.86	0.008	3.73	0.002	0.068	0.084	90.245
Mechanical properties									
Flow resistance (Mpa)			Tensile strength (Mpa)				Elongation (%)		
865			937				11		

**Table 2:** Chemical composition of Hank’s biological solution.

Nr. Crt	Chemical Compound	Concentration g/L
1	NaCl	8.8
2	KCL	0.4
3	CaCl_2_·2H_2_O	0.35
4	Na_2_HPO_4_·H_2_O	0.25
5	MgCl_2_	0.19
6	MgSO_4_·7H_2_O	0.06
7	C_6_H_12_O_6_	1

**Table 3:** Physicochemical characteristics of Hank’s biological solution.

Electrolyte	pH	Conductivity (mS/cm)	Salinity (ppt)
Hank	7.41	14.6	8.4

## Data Availability

Not applicable.
